# Developing discriminate model and comparative analysis of differentially expressed genes and pathways for bloodstream samples of diabetes mellitus type 2

**DOI:** 10.1186/1471-2105-15-S17-S5

**Published:** 2014-12-16

**Authors:** Chang Liu, Lili Lu, Quan Kong, Yan Li, Haihua Wu, William Yang, Shandan Xu, Xinyu Yang, Xiaolei Song, Jack Y Yang, Mary Qu Yang, Youping Deng

**Affiliations:** 1 Wuhan University of Science and Technology, Wuhan, China; 2 Rush University Cancer Center, and Departments of Internal Medicine and Biochemistry, Rush University Medical Center, Chicago, IL, USA; 3 Guangxi Armed Police General Hospital, Nanning, Guangxi Autonomous Region, China; 4 Department of Computer Science, George W. Donaghey College of Engineering and Information Technology, University of Arkansas at Little Rock, 2801 S. University Avenue, Little Rock, Arkansas 72204, USA; 5 National 48610 Hospital of Armed Police Force, Panjin, Liaoning Province, China; 6 Division of Biostatistics and Biomathematics, Massachusetts General Hospital and Harvard Medical School. Boston, MA 02114, USA; 7 Center for Computational Biology and Bioinformatics, Indiana University School of Medicine. Indiana University Purdue University Indianapolis, Indiana 46202, USA; 8 MidSouth Bioinformatics Center, Department of Information Science, George W. Donaghey College of Engineering and Information Technology, University of Arkansas at Little Rock, 2801 S. University Avenue, Little Rock, Arkansas 72204, USA; 9 Joint Bioinformatics Graduate Program, University of Arkansas at Little Rock and University of Arkansas for Medical Sciences, Little Rock, Arkansas 72204, USA

**Keywords:** T2D, pre-diabetic, differential gene expression, Gene Ontology (GO) analysis, Discriminant model

## Abstract

**Background:**

Diabetes mellitus of type 2 (T2D), also known as noninsulin-dependent diabetes mellitus (NIDDM) or adult-onset diabetes, is a common disease. It is estimated that more than 300 million people worldwide suffer from T2D. In this study, we investigated the T2D, pre-diabetic and healthy human (no diabetes) bloodstream samples using genomic, genealogical, and phonemic information. We identified differentially expressed genes and pathways. The study has provided deeper insights into the development of T2D, and provided useful information for further effective prevention and treatment of the disease.

**Results:**

A total of 142 bloodstream samples were collected, including 47 healthy humans, 22 pre-diabetic and 73 T2D patients. Whole genome scale gene expression profiles were obtained using the Agilent Oligo chips that contain over 20,000 human genes. We identified 79 significantly differentially expressed genes that have fold change ≥ 2. We mapped those genes and pinpointed locations of those genes on human chromosomes. Amongst them, 3 genes were not mapped well on the human genome, but the rest of 76 differentially expressed genes were well mapped on the human genome. We found that most abundant differentially expressed genes are on chromosome one, which contains 9 of those genes, followed by chromosome two that contains 7 of the 76 differentially expressed genes. We performed gene ontology (GO) functional analysis of those 79 differentially expressed genes and found that genes involve in the regulation of cell proliferation were among most common pathways related to T2D. The expression of the 79 genes was combined with clinical information that includes age, sex, and race to construct an optimal discriminant model. The overall performance of the model reached 95.1% accuracy, with 91.5% accuracy on identifying healthy humans, 100% accuracy on pre-diabetic patients and 95.9% accuract on T2D patients. The higher performance on identifying pre-diabetic patients was resulted from more significant changes of gene expressions among this particular group of humans, which implicated that patients were having profound genetic changes towards disease development.

**Conclusion:**

Differentially expressed genes were distributed across chromosomes, and are more abundant on chromosomes 1 and 2 than the rest of the human genome. We found that regulation of cell proliferation actually plays an important role in the T2D disease development. The predictive model developed in this study has utilized the 79 significant genes in combination with age, sex, and racial information to distinguish pre-diabetic, T2D, and healthy humans. The study not only has provided deeper understanding of the disease molecular mechanisms but also useful information for pathway analysis and effective drug target identification.

## Introduction

Diabetes mellitus of type 2 (T2D) is among 10 most common diseases. According to World Health Organization (WHO), it is estimated that 347 million people suffer from type 2 diabetes. T2D is usually considered not reversible, but if not controlled well, it will eventually lead to fatal complications. However, earlier diagnosis of diabetes requires preventative screening and regular healthcare monitoring, which are not always provided in many countries. Therefore, lower income countries have higher death rates from diabetes. WHO estimated that diabetes deaths will be doubled by the year of 2030. Unfortunately about half of diabetes patients do not realize that they have the disease [1]. In United States of America, diabetes commonly occurs in all ethnic population, and is the seventh most common cause of death. According to American Diabetes Association, the prevalence of the disease is now climbing towards 10% and 30 million people. T2D is also an age related disease. The prevalence in seniors is more than a quarter of the entire population. Because T2D is related to lifestyles, especially foods and exercises, the disease has been and will be consistently increasing and has been estimated to reach one third of the population by 2050 [2]. While type 1 diabetes mellitus is known as juvenile diabetes with strong genetic dispositions or viral involvement, type 2 diabetes is known genetically diverse and contribute to more than 90% of all diabetes [3]. All diabetes can lead to not only long-term but also lethal complications, including cardiovascular, retina, nerve system complication, chronic renal failure, and greater susceptibility of infection. Because T2D is so popular, it has led to the increasing death rate as well as social and economic burdens. While T2D has been known as a genetically complex and multifactorial disease with impaired glucose regulation, such as impaired fasting glucose (IFG) and impaired glucose tolerance (IGT), it is a gradually developing disease and commonly considered as irreversible at our current treatment capabilities. T2D often proceed gradually with aging and can be worsened by many factors, such as hypertension, high cholesterol, lacking of exercise, genetic disposition and family history of diabetes [4-7]. Obesity and immune/inflammatory issues can contribute to the disease [8,9]. The changes of modern lifestyles in humans have been considered an important factor in the ever-increasing T2D occurrences.

While so far no effective treatment can virtually cure diabetes, significant research progresses have been made in understanding the genetic changes in the development of T2D, especially the mechanisms of gene regulation. New research has led to better prevention of the development of T2D and effective identification of drug targets for blocking or even reversing the disease development [10]. Frayling from the UK's Peninsula College of Medicine and Dentistry found that Single Nucleotide Polymorphism in fat mass and obesity associated gene (FTO) has a strong association with the risk of T2D [11]. Zhao's research showed that analysis of combined gene expression and lipid profiles helped to identify the pathogenesis of T2D [12]. As our research has been unfolded to the study of differential expression of genes and regulatory mechanisms of the genes in diabetes mellitus, we have made significant progresses in finding specific genes and the specific pathways to be targeted by drugs for the purposes of preventing and inversing the disease development.

Pre-diabetic or early stage T2D patients usually do not have any noticeable symptoms and are not diagnosable without blood analysis. Clinical diagnosis of diabetes includes fasting plasma glucose test [13], hemoglobin A1c test, glucose tolerance test, and clinical screening during physical checkup, but all rely on laboratory blood analysis [14]. Although blood analysis does not provide any genetic, genomic or pathological information about the disease, such information can be useful in assessing the stage, subtype, prognosis, damage and impact of the disease. New research efforts include differential gene expression profiling and genome-wide association studies (GWAS) [15-19] have been made. Yet, the molecular mechanisms of disease are potentially heterogeneous while the limited availability of samples for genome-wide association studies almost prevented effective population genetics analysis and sub-type identification of the disease. In this paper, we assessed and analyzed a number of patients and healthy humans using genomic information including peripheral white blood cell gene expression profile (GEP), and phonemic information including age, gender, and race. Our past studies found that phonemic factors have little influence on GEP. Hierarchical clustering and principal component analysis (PCA) showed that GEP were not directly related to the phonemic factors including gender, blood sugar level, age, and race. However, race and gender are not randomly distributed in the clustering analysis, which implicated that they had potential relevancy with GEP [20]. Therefore, we use age, gender, race, and 79 significant genes as parameters to derive the discriminant model. The model was used successfully to classify the samples of different disease stages respectively with high performance and accuracy. This has led to find molecular mechanisms and genetic diversity for identifying sub-types and pathogenesis of type 2 diabetes mellitus.

## Materials and methods

### Research objectives, and laboratory and clinical data

Based on our previous research, we now aim to identify characteristic genes and pathways in diabetes. The criteria for the diagnosis of T2D were based on the American Diabetes Association (ADA) [21] guidelines in accordance with the symptoms in diabetes. The diagnostic criteria are positively correlated with body mass index (BMI) and fasting blood glucose level > 126 mg/dl, or 2H blood glucose level > 200 mg/dl in the oral glucose tolerance test. A total of 142 bloodstream samples were collected, including 47 people from a healthy control group, 22 pre-diabetic, and 73 T2D patients. The experiments were carried out and analyzed using comprehensive information that includes age, gender, race, and GEP. Tougaloo College in Mississippi provided data, and the research was approved by the Institutional Review Board of Tougaloo.

### RNA isolation

Firstly, Total RNA from 8 - 10 ml of peripheral blood white cells was extracted according to the manufacturer's instructions with LeukoLock ™ general RNA system (Anbion Inc, Austen, Texas, USA). Then the content of the RNA was detected and separated by using Nanodrop spectrophotometer and Agilent 2100 Bioanalyzer (Agilent Technologies, Santa Clara, California, USA). All protocols have been carefully assured and all RNAs have been carefully inspected to ensure that RNAs were not degenerated.

### Microarray experiments

All standard protocols and instructions on handling RNA have been carefully followed. A total of 500 ng of RNA was amplified and labeled by Agilent Low RNA Input Fluorescent Linear Amplification. 850 ng of Cy5- (universal control) and Cy3-labeled (sample) cRNA were mixed and dispersed by the Agilent *In Situ *Hybridization Kit for every two color array. According to the Agilent 60-mer oligo microarray, hybridizations were put forward for 17 hours in rotating hybridization oven first, and then an Agilent Scanner (G2565AA, Agilent Technologies, Wilmington, Delaware, USA) was used to wash and scan them. Finally, quality control analysis was performed and the Agilent Feature Extraction software (v.9.5.3.1) was used to handle the information from the chips and correction of background noises from default parameters.

### Microarray data analysis

Gene chip data was analyzed using GeneSpring 10.0 and quality control was conducted with Pearson correlation coefficients between each sample of the experiment and others pair-wisely. Samples showing less than 80% of correlation with other samples were excluded for further analysis. Scanning probe intensity of less than 5 was directly converted into 5. All probe values using the chip in the 5000 percentile were standardized as per-chip (inner) data. Each gene was standardized using the median value of one gene in all of the samples. The probe characteristics were screened by markers while "Occupying" or "Absence" of the symbol could be used to define the Aglient properties. The Database for Annotation, Visualization and Integrated Discovery (DAVID, http://david.abcc.ncifcrf.gov/) online analysis tool was used to analyze chromosomal localization and function of differentially expressed genes.

### Statistical analyses

#### Analysis of differentially expressed genes

GeneSpring GX 10.0 (Agilent Technologies, Santa Clara, California, USA) software was used for gene expression analysis. To standardize the data using the method of Lowess [22], each chip used the 50% as the base, and each gene was standardized by a median reference. The standardized sample data was entered into the GeneSpring GX software. Firstly, quality controls were performed to select the data and samples of the inserted microarray data. Secondly, all the samples were divided into 3 groups: the normal group (ND), pre-diabetic group (PD), and T2D group (D). Thirdly, matched pairwise analysis was conducted and groups were compared pair-wisely. Finally, less significant genes were filtered out. The selection threshold was set as: False discovery rate (FDR) = 5%, p < 0. 05, |fold change| ≥ 2.

#### Discriminate analysis

Fisher's exact test was used as a discriminant method and a discriminant model was built by all parameters in the experimental group. Statistical software package SPSS 16.0 was used. All samples were finally classified by the discriminant model.

## Results

### Quality control on samples and entities

**Figure 1:**
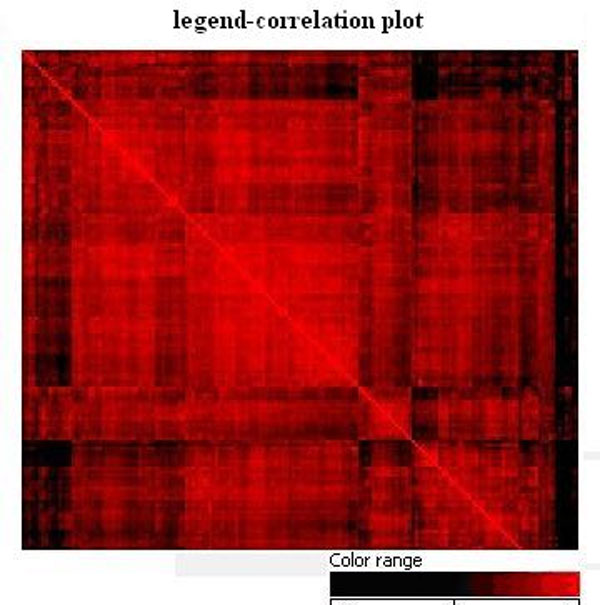
**Correlation plot shows the Pearson correlation coefficient for each pair of array and displays in visual form as heatmap**. Red color means highly correlated, black color means no or barely any correlation.

We performed quality control analysis. Figure 1 showed that the gene chip quality was well controlled and acceptable. The correlation coefficients were greater than 0.9. The correlation plot shows the Pearson correlation coefficient for each pair of array and displays in visual form as a heatmap. The correlation coefficient is a number between 0 and 1. If there is no relationship between the samples, the correlation coefficient is zero or very low, while high correlation gives a coefficient value close to and up to1.0 (Figure 1). Thus, it appears the higher the correlation coefficient, the better quality of the data.

### Hierarchical clustering

**Figure 2:**
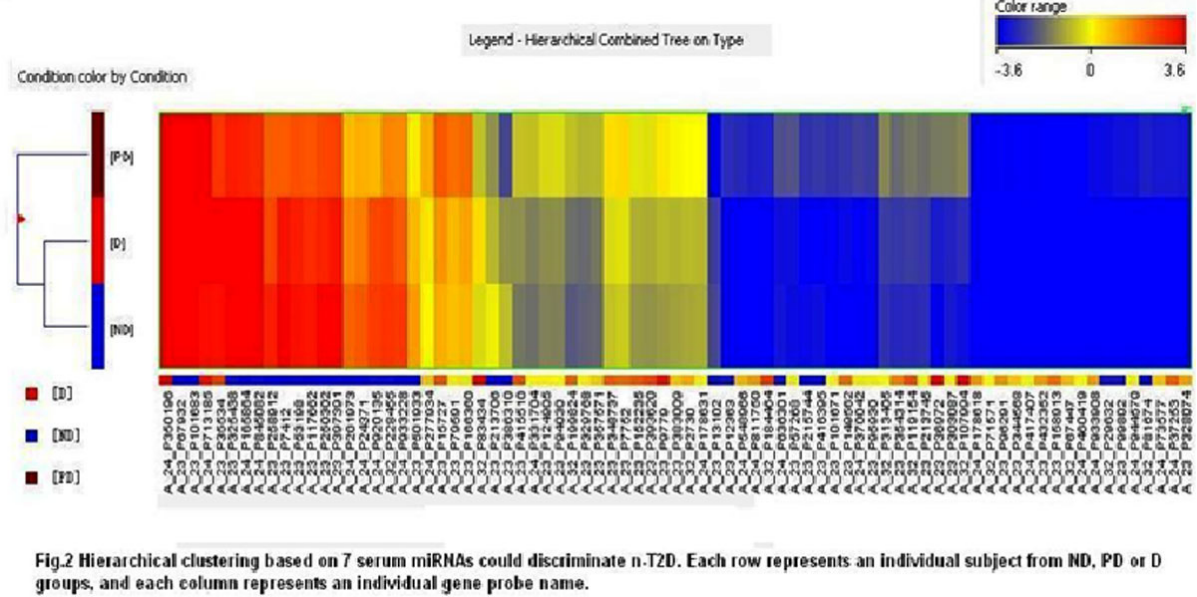
**Hierarchical clustering based on 7 serum miRNAs that could discriminate T2D, pre-diabetes and normal samples**. Columns represent names of individual gene probes while rows represent samples from T2D (D), pre-diabetic (PD) and non-diabetic (ND) humans.

Hierarchical clustering analysis was performed on all samples of diabetes, pre-diabetes, and normal human groups as shown in Figure 2. PD group and the other two groups are clearly separated. The results showed that the gene expression levels of PD group from GEP were relatively most significant compared to the other two groups (Figure 2).

### Differential genes

We identified 79 significantly differentially expressed genes with fold change ≥ 2.0. In the differentially expressed gene analysis between the pre-diabetes, and T2D groups, there are 24 genes with fold change ≥ 2.0, among which 7 genes were up-regulated and 14 genes were down-regulated. In the comparison between pre-diabetes and normal human groups, there are 74 genes with fold change ≥ 2.0, among which 20 genes were up-regulated and 14 genes were down-regulated. A number of genes were expressed differentially among all groups. For example TFEB gene was not only significant in the T2D and pre-diabetes comparison but also in the healthy and pre-diabetes comparison, with 2.76 and 3.88 times in difference in each pairs of comparisons respectively.

### Chromosome location of differentially expressed genes

We performed bioinformatics analysis, and found 79 differentially expressed genes with more than 2 fold changes. Locations of 3 of the79 genes are not known and the other 76 differentially expressed genes were distributed across different chromosomes. Chromosome 1 contains 9 of them (11.3%); and chromosome 2 contains 7 of them (8.86%). These two chromosomes are most abundant in significant genes. No differentially expressed genes were found in chromosomes 4, 9, 20, and the sex Y chromosome. It is reasonable that T2D does not have obvious gender difference. 57 genes were located in the long arm of a chromosome, accounting for 72.2% in total; 19 genes were located in the short arm of a chromosome, accounting for 24.1%. Although chromosome 1 is the longest chromosome in a human genome, differentially expressed genes are still more abundant relatively on chromosomes 1 and 2.

### Functional classifications of differentially expressed genes

We performed gene ontology (GO) analysis and classified differentially expressed genes by biological pathways. 11 significant genes were found in the regulation of cell proliferation process, 5 genes were found in taxis process, 5 genes were found in chemotaxis process, 7 genes were found in positive regulation of cell proliferation process, 3 genes existed in sperm motility process and T2D may impact on male sexual functionality. We found 6 significant genes in the localization of cell process, 6 genes in cell motility process, 7 genes in cell motion process, 3 genes in rho protein signal transduction process, 3 genes in rho protein signal transduction process, 5 genes in epithelium development process, 8 genes in cell adhesion process, 8 genes in biological adhesion process, 5 genes in locomotory behavior process, 3 genes in cellular defense response process which implicate that T2D may impact cellular function. For examples, increased B-cell proliferation has been known in pre-diabetes and implicates that the pancreas may have loosen hormone secretion function. Impaired neutrophil chemotaxis has been known in diabetic patients. Furthermore we found 6 genes in behavior process, 13 genes in cell surface receptor linked signal transduction process, 4 genes in tissue morphogenesis process, 5 genes in negative regulation of cell proliferation process, 4 genes in tube development process, and 3 genes existed in morphogenesis of an epithelium process. Results are summarized in Table 1. The Gene Ontology analysis has provided deeper insights into the molecular mechanisms of T2D that can help the identification of drug targets in blocking the pathways in the disease development.

**Table 1:** Classification of Biological Process Categories Based on Gene Ontology (GO) analysis.

Biological Process	Genes	P-value	Count
Regulation of cell proliferation	LAMB1, ALDH1A2, GPNMB, PTN, IL8RB, CXADR, CTTNBP2, PRAME, IL4, IGFBP5, LAMA1, CXADR	0	11
Taxis	CCR3, IL8RB, CXCL14, IL4, IL8RA	0.002	5
Chemotaxis	CCR3, IL8RB, CXCL14, IL4, IL8RA	0.002	5
Postive regulation of cell proliferation	LAMB1, ALDH1A2, PTN, IL8RB, PRAME, IL4, LAMA1	0.003	7
Sperm motility	LAMA1, APOB, CTTNBP2	0.004	3
Localization of cell	ROPN1B, APOB, NR2F1, IL8RB, CTTNBP2, LAMA1	0.004	6
Cell motility	ROPN1B, APOB, NR2F1, IL8RB, CTTNBP2, LAMA1	0.004	6
Cell motion	ROPN1B, APOB, NR2F1, IL8RB, SEMA6A, CTTNBP2, LAMA1	0.006	7
Rho protein signal transduction	ROPN1B, COL1A2, ARHGAP29	0.008	3
Epithelium development	AHNAK, ALDH1A2, ZIC2, NEUROG3, LAMA1	0.008	5
Cell adhesion	LAMB1, ROPN1B, CCR3, FLRT2, GPNMB, BCAN, CXADR, LAMA1, CXADR	0.011	8
Biological adhesion	LAMB1, ROPN1B, CCR3, FLRT2, GPNMB, BCAN, CXADR, LAMA1, CXADR	0.011	8
Locomotory behavior	CCR3, IL8RB, CXCL14, IL4, IL8RA	0.016	5
Cellular defense response	CCR3, IL8RB, IL4	0.02	3
Behavior	CCR3, PTN, IL8RB, CXCL14, IL4, IL8RA	0.025	6
Cell surface receptor linked signal transduction	P2RY14, CCR3, COL1A2, SEMA6A, IL8RB, PTN, IL8RA, LAMA1, FFAR2, ROR1, GPR161, HRH4, STC2	0.026	13
Tissue morphogenesis	ALDH1A2, COL1A2, ZIC2, LAMA1	0.026	4
Negative regulation of cell proliferation	ALDH1A2, GPNMB, CXADR, CTTNBP2, IGFBP5, CXADR	0.039	5
Tube development	ALDH1A2, SALL1, CTTNBP2, ZIC2	0.043	4
Morphogenesis of an epithelium	ALDH1A2, ZIC2, LAMA1	0.05	3

### Discriminate analysis

A discriminant model was built by 82 parameters (V2-V83) from the 142 samples, and V60 was eliminated in the analysis process. Two typical discrimination functions (Function1, Function2) were extracted, among which Function1 explained 74.4% of all variations and Function2 explained the rest 25.6%. Testing results showed a p-value = 0.000 from Function1 through Function2, which meant the discriminate function has the greatest statistical significance. The functional expressions of discriminate functions were analyzed.

**Figure 3:**
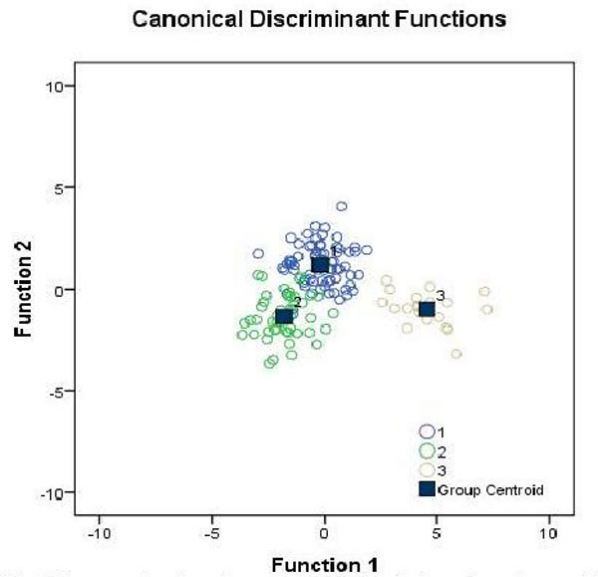
**Canonical discriminant functions**.

The performance of classification results from the discriminant model derived by principles of back substitution was high. Results showed that 95.1% samples were correctly classified overall, and groups of pre-diabetes were all correctly predicted. The accuracy rate of T2D group was 95.9% and the accuracy rate of healthy group was 91.5%. The results are summarized in Table 2. Figure 3 presented a graph of scattered discriminant scores. It can be seen from the Figures 1, 2, 3 that the model can distinguish disease group from health group, especially the distinctions between pre-diabetes group and others were particularly obvious. Therefore, the model built in this study can provide useful information for early biomarker identification of the disease.

**Table 2:** Classification Results

			Predicted Group Membership			
				
		VAR00001	D		PD	Total
Original	Count	D	70(95.9%)	3(4.1%)	0(0%)	73
%		ND	4(8.5%)	43(91.5%)	0(0%)	47
		PD	0(0%)	0(0%)	22(100%)	22

a. 95.1% of original grouped cases correctly classified

## Discussion

While Grayson et al. [23] published an article about the study of human peripheral blood gene chips, their research only showed that the difference of gene expression in T2D played an important role in signal transduction of T cell activation, but the number of samples used in their study (only six cases) was limited, and their samples did not include racial information. When Lei Kong et. al. [10] discussed the significance of seven microRNA in the serum (miR-9, miR-29a, miR-30d, miR34a, miR-124a, miR146a, and miR375) that are related to diabetes, their samples were 56 healthy controls, 18 newly diagnosed T2D patients (n-T2D), and 19 pre-diabetic patients with known susceptibilities (s-NGT). Canonical discriminant analysis results confirmed 70.6% of n-T2D samples (12/17), while the samples of the s-NGT and pre-diabetic could not be distinguished from each other. Rui Wang-Sattler et al [24] quantified 140 metabolites of fasting serum samples of 4297 and confirmed candidate biomarkers for pre-diabetes using metabolomic methods to identify three metabolites [glycine, lysophosphatidylcholine (LPC) and acetyl] for prediction of IGT and T2D. Wang et al. [25] studied the samples of 189 T2D and showed that the increasing content of a small group of essential amino acids [leucine (Leu), valine (Val), isoleucine (Ile)] and aromatic amino acids [phenylalanine (Phe), tyrosine (Tyr)] in serum are associated with risk of T2D by five-fold increment. Our study provides complementary insights into the mechanism of T2D and useful information for better prevention and treatment of the disease and effective identification of drug targets.

## Conclusion

This study identified 79 significant genes with more than 2-fold changes in differentially expressed genes using bioinformatics approaches. Differentially expressed genes were mainly distributed in chromosomes 1, 2, 3, 5, and 7, with more abundance in chromosomes 1 and 2. According to Gene Ontology and gene functional analysis, genes which belong to the regulation of cell proliferation were very significant and played important roles in the pathogenesis of T2D. Many genes have multiple functions. For instance, insulin receptor is involved in diabetes and also plays a role in cell proliferation and cancer. Diabetes is a disorder of metabolic syndrome, which will also induce cell proliferation changes on some tissues. T2D patients may have compromised cellular function in absorbing bloodstream sugar, it is reasonable to have elevated gene expression relating to cell proliferation pathways. This study discussed feasibility of combined molecular and bioinformatics methods to distinguish normal humans, pre-diabetic, and T2D effectively. We have analyzed 142 blood samples, including the healthy control group of 47 people, 22 pre-diabetic, and 73 T2D patients. By comparing the gene chip spectrum of these samples, T2D biomarkers can be implicated from the 79 significant genes. Discriminant analysis model showed that combination of 79 genes with three phonemic factors could effectively distinguish healthy human, pre-diabetic, and T2D patients. The results showed that 95.1% of the samples were correctly classified, amongst which 100% was acheived in predicting pre-diabetic samples, 95.9% accuracy was achieved in T2D group, and 91.5% accuracy in healthy human group. The research provided a combined molecular and pedigree analytic method that could potentially lead to an effective screening tool for identifying overall health or illness of humans and prediction of the prognosis of the disease development. The results also showed that 79 genes are significant in diabetes, and these 79 differentially expressed genes have revealed deeper molecular mechanisms of the disease. The research has also led to effective pathway and drug target identification, treatment planning and future therapeutic strategies. In addition, since the discriminant analysis method can separate the pre-diabetes group well from the other two groups, it can lead to the development of new diagnostic tool for the earlier detection of the disease.

## Competing interests

The authors declare that they have no competing interests.

## Authors' contributions

YD and MQY conceived the project; YD and CL designed and coordinated the study. CL, YL, SX, QK ZW, WY and MQY performed the experiments and analyzed the data. LL, HW, XS, XY, and JYY participated in array data analyses. CL and WY summarized the results and drafted the manuscript. WY and YD revised and finalized the manuscript which was read and approved by all authors.
